# Comparative study of sulfite pretreatments for robust enzymatic saccharification of corn cob residue

**DOI:** 10.1186/1754-6834-5-87

**Published:** 2012-12-04

**Authors:** Lingxi Bu, Yang Xing, Hailong Yu, Yuxia Gao, Jianxin Jiang

**Affiliations:** 1 Department of Chemistry and Chemical Engineering, Beijing Forestry University, Beijing 100083, China

**Keywords:** Sulfite pretreatment, Enzymatic hydrolysis, Corn cob residue, Sulfonic group, Conductometric titrations

## Abstract

**Background:**

Corn cob residue (CCR) is a kind of waste lignocellulosic material with enormous potential for bioethanol production. The moderated sulphite processes were used to enhance the hydrophily of the material by sulfonation and hydrolysis. The composition, FT-IR spectra, and conductometric titrations of the pretreated materials were measured to characterize variations of the CCR in different sulfite pretreated environments. And the objective of this study is to compare the saccharification rate and yield of the samples caused by these variations.

**Results:**

It was found that the lignin in the CCR (43.2%) had reduced to 37.8%, 38.0%, 35.9%, and 35.5% after the sulfite pretreatment in neutral, acidic, alkaline, and ethanol environments, respectively. The sulfite pretreatments enhanced the glucose yield of the CCR. Moreover, the ethanol sulfite sample had the highest glucose yield (81.2%, based on the cellulose in the treated sample) among the saccharification samples, which was over 10% higher than that of the raw material (70.6%). More sulfonic groups and weak acid groups were produced during the sulfite pretreatments. Meanwhile, the ethanol sulfite treated sample had the highest sulfonic group (0.103 mmol/g) and weak acid groups (1.85 mmol/g) in all sulfite treated samples. In FT-IR spectra, the variation of bands at 1168 and 1190 cm^-1^ confirmed lignin sulfonation during sulfite pretreatment. The disappearance of the band at 1458 cm^-1^ implied the methoxyl on lignin had been removed during the sulfite pretreatments.

**Conclusions:**

It can be concluded that the lignin in the CCR can be degraded and sulfonated during the sulfite pretreatments. The pretreatments improve the hydrophility of the samples because of the increase in sulfonic group and weak acid groups, which enhances the glucose yield of the material. The ethanol sulfite pretreatment is the best method for lignin removal and with the highest glucose yield.

## Background

Waste lignocellulosic material, which is easily available, inexpensive, and renewable, represents a kind of significant cellulosic biomass as raw material to produce fuel ethanol with many advantages in bioethanol conversion [1]. Corn cob residue (CCR) is a kind of waste lignocellulosic material. During the production of furfural from the lignocellulosic materials with abundant pentose sugars, such as corncob, the hemicelluloses have been hydrolyzed to furfural in a dilute acid environment at high temperature, leaving the lignin and cellulose in the CCR [2]. It has been estimated that about 12–15 tons of CCR can be obtained after 1 ton of furfural is produced, and an average of 23 million tons of CCR were available annually for alternative use in China [3]. However, the residue, considered as waste, are widely utilized for burning at present, far away form resource utilization. It would be a better choice to produce bioethanol with such abundant lignocellulosic waste.

Among the available technologies for lignocelluloses-to-ethanol production, a conversion process based on enzymatic hydrolysis is considered the most promising for large-scale operation [4,5]. However, one of the key factors to construct the recalcitrance of lignocellulosic biomass is the presence of lignin, which plays the “glue” to bind cellulose and hemicellulose. Besides playing a physical barrier, lignin has also been found to irreversibly adsorb enzymes, which causes enzyme loss and decrease in the saccharification rate [6]. Therefore, delignification is always adopted to overcome the recalcitrance of lignocellulosic biomass and increase the enzymatic digestibility of cellulose.

The effect of lignin content on enzymatic hydrolysis of CCR has been evaluated, and it is found that the glucose yield was improved by increasing the lignin removal. However, the maximum glucose yield of CCR was obtained when the residue with a lignin content of about 21.0% [3]. The results further prove that the chemical and physical structure of lignin plays a significant role in determining the magnitude of inhibition of lignin to hydrolysis. There has been strong evidence [7] supporting the role of hydrophilic interactions in the non-productive binding of cellulases to lignin. Multiple studies [7,8] have shown that the addition of the surfactant to cellulolytic hydrolysis improved hydrolysis yields. It reported that increasing the carboxylic acid content of the lignin seemed to significantly decrease the non-productive binding of cellulase and consequently increased the enzymatic hydrolysis of the cellulose [9]. So the hydrolysis yields of CCR may be benefited from the enhanced hydrophily of lignin after a temperate pretreatment.

The sulfite process has been used for pretreating wood chips for ethanol production. Sulfonation of lignin increases its hydrophilicity, which will promote the enzymatic hydrolysis process [10,11]. And the lignosulfonate has been used as pesticide emulsifier, oil field chemicals, dyeing and finishing auxiliaries for textile, which can been obtained from the concentrated sulfite pretreated solution. Traditional sulfite pulping has been in industry practice for more than a century and can be operated over a wide range of pH and temperature. And the active reagents in sulfite pretreatment liquor are also depended on the pH of the pretreatment temperature [12]. Sulfonation is always enhanced because of the acid or alkaline catalysis. The acid sulfite and neutral sulfite pretreatment has been well documented as the SPORL pretreatment [12] with numerous publications to variety of feed stocks. And sulfite pretreated in alkaline environments also can increase the sulfonation and dissolubility of lignin. It has reported that during fraction of *spruce* by SO_2_-ethanol-water treatment, lignin is effectively dissolved, whereas cellulose is preserved in the solid (fiber) phase [13]. And the organophilic sulfite pretreatment is also a good choice for lignin separation and sulfonation because of the addition of ethanol, which caused a reduction of the surface tension and a benefit of solution penetration. Moreover, the hydrolysed lignin can be dissolved and recovered in the organophilic phase to obtained high purity lignin.

Our previous study has found that the glucan in CCR was easily degraded in severe pretreated processes. So in this study, the CCR were pretreated with sodium sulfite under moderate condition in acidic, alkaline, neutral, and ethanol environments to enhance the hydrophily of lignin by sulfonation reaction. And the objective is to compare the composition and characteristic variation of CCR during these sulfite pretreatments, and to compare the differences of saccharification rate and yield caused by these variations of the samples.

## Results and discussion

### Chemical composition of substrates

The variation of the chemical composition in CCR is given in Table 1. Glucan (48.1%) and lignin (43.2%) accounted for more than 90% of the CCR, implying that they are the main chemical compositions of the CCR and the hemicelluloses have been mostly removed during furfural production. After the pretreatments, lignin in the CCR had been removed partly, resulting in an increase in glucan proportion. Comparatively speaking, the amount of removed lignin after alkaline and ethanol sulfite pretreatment was higher than that after acidic and neutral sulfite pretreatment. The proportion of lignin in samples after alkaline and ethanol sulfite pretreatment decreased to 35.9% and 35.5%, respectively, which were lower than that in the samples after acidic pretreatment (38.0%) and neutral sulfite pretreatment (37.8%). Moreover, the residual lignin in the treated samples, based on the quality of the untreated CCR, was in according with the results of lignin proportion in pretreated samples. The lowest residual lignin sample was from the alkaline sulfite pretreated sample, only 28.08%.

**Table 1:** Variation of corn cob residue chemical composition after sulfite pretreatments

**Component/%**	**RM **^**a**^	**Neutral **^**a**^	**Acidic **^**a**^	**Alkaline **^**a**^	**Ethanol **^**a**^
Solid Yield	—	89.23	91.85	78.18	84.68
Glucan	48.10±0.306	51.55±0.273	49.31±0.363	51.77±0.369	55.51±0.391
Klason Lignin	41.58±0.296	37.35±0.259	37.29±0.175	35.57±0.291	35.14±0.251
Acid Soluble Lignin	1.61±0.084	0.44±0.017	0.65±0.012	0.354±0.011	0.385±0.009
Total Lignin	43.19±0.306	37.79±0.352	37.95±0.172	35.92±0.280	35.53±0.243
Ash	6.84±0.211	7.95±0.358	8.51±0.139	9.21±0.326	7.91±0.199
Residual Glucan ^b^	-	45.99	45.29	40.47	47.01
Residual Lignin ^b^	-	33.72	34.86	28.08	30.09

Note: ^a^: RM represents the raw material of corn cob residue, while Neutral, Acidic, Alkaline, and Ethanol represent corn cob residue treated with sulfite in neutral, acidic, alkaline, and ethanol environments, respectively. ^b^: based on the quality of the untreated corn cob residue.

The variation of glucan proportion after sulfite pretreatments was not the same as the decrease in lignin proportion. The sample treated with ethanol sulfite had the highest glucan proportion (55.5%), while the values of the neutral and alkaline sulfite pretreated sample were 51.6% and 51.8%, respectively. The sample of acidic sulfite sample had the lowest glucan proportion (49.3%) of all the pretreated samples. However, the residual glucan in all the treated samples had endured a decline, especially the alkaline sulfite pretreated sample (40.47%). The ethanol sulfite pretreated sample had the highest residual glucan (47.01%), a little lower than the glucan proportion in raw material (48.10%).

The ratio of ash increased as the degradation of organic matter (including glucan and lignin) during sulfite pretreatments. The fact that acid soluble lignin in the CCR decreased after sulfite pretreatments was attributed to the solvable lignin with low molecular weight during pretreated process.

Undoubtedly, during sulfite processes, the delignification was realized by the formation of soluble fragments after lignin sulfonation and degradation. The considerable distinctive composition of pretreated samples was attributed to the different active reagents in the sulfite liquor, which depended on its pH and temperature [12,14]. The nucleophilic reaction of these active reagents resulted in the sulfonation and degradation of lignin in the raw materials [15]. The lignin solubility is connected with the pH value of the pretreated solution. The alkaline solution has better lignin solubility at a higher pH value than the acidic solution with a lower pH value [16]. So, the amount of lignin removal after alkaline pretreatment was more than that after treatment with acidic and neutral sulfite. During the ethanol sulfite pretreatment, the surface tension reduced because of the addition of ethanol, which was a benefit of solution penetration and lignin sulfonation, resulting in more lignin removal [17-19]. Moreover, the boiling point of ethanol is lower than that of water; hence, the pressure in the ethanol sulfite pretreated bottle was the highest. These advantages of ethanol sulfite pretreatment enhanced the sulfonation and degradation of lignin. The dissolved lignin can be directly used in various industrial fields as the surfactant after the concentration. It paved a new path for the utilization of the lignin in CCR.

It cannot be neglected that the cellulose can be degraded at acidic or alkaline solution [20,21]. Because of the acid hydrolysis of cellulose during acidic sulfite pretreatment, the increase in the glucan proportion was the lowest in all sulfite pretreatments. However, the lowest yield and the highest ash amount in the CCR after alkaline sulfite pretreatment was attributed to degradation of organic polymer, not only the removal of lignin, but also the damage of cellulose. The results of residual glucan also supposed these conclusions. However, it can not be neglected that the ethanol sulfite pretreatment had the weakest glucan degradation among these pretreatments. Moreover, the strong vitality of this organic pretreatment is also reflected in the cyclic utilization of ethanol and high purity lignin obtained from the pretreated solution.

### Enzymatic saccharification

**Figure 1:**
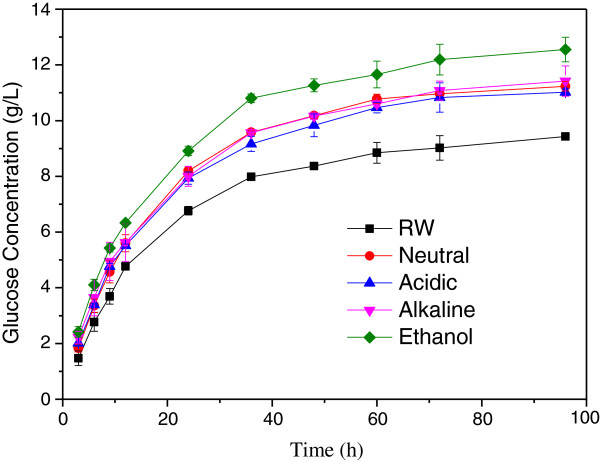
**Glucose concentration variation of corn cob residue after sulfite pretreatments.** RM represents the raw material of corn cob residue, while Neutral, Acidic, Alkaline and Ethanol represent corn cob residue treated with sulfite in neutral, acidic, alkaline and ethanol environment, respectively.

The glucose concentration of the pretreated CCR was higher than that of the raw CCR (Figure 1). The glucose released from the substrates was increased rapidly in the initial stage, while the rate of hydrolysis progressively reduced as the reaction proceeded. The glucose concentration in the raw material saccharification solution reached 6.76 g/L in the first 24 h, which was over 50% of the concentration at 96 h (9.43 g/L). The sulfite pretreated samples shared the same tendency with the CCR without pretreatment, but the glucose concentration in the saccharification solution of these samples was higher than that in the CCR at corresponding hours. After 96 h of enzymatic hydrolysis, the glucose concentration in the ethanol sulfite pretreated sample was the highest (12.56 g/L), and the glucose concentration in the other three samples had approximate values (11.23 g/L, Neutral; 11.02 g/L, Acidic; 11.42 g/L, Alkaline), which were higher than that of the untreated sample.

**Figure 2:**
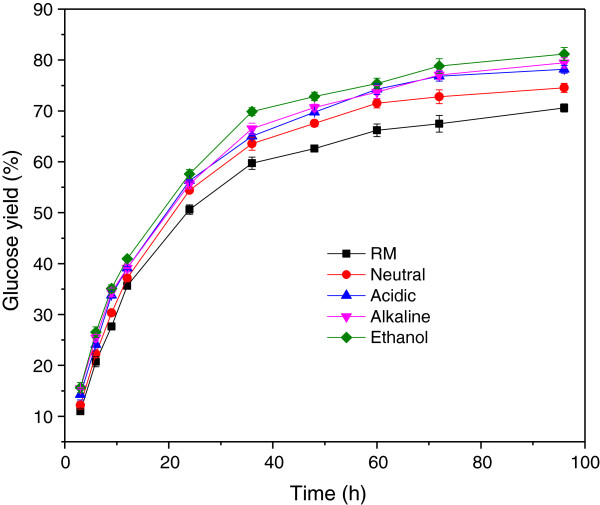
**Glucose yield variation of corn cob residue after sulfite pretreatments.** RM represents the raw material of corn cob residue, while Neutral, Acidic, Alkaline and Ethanol represent corn cob residue treated with sulfite in neutral, acidic, alkaline and ethanol environment, respectively.

Similarly, the glucose yield (based on the cellulose in the treated sample) of all the samples experienced a rapid growth in the first 24 h, and the growth moderated from then on (Figure 2). And, the glucose yield of the samples after sulfite pretreated was higher than that of untreated CCR. However, as the glucan proportion of the samples was not the same, the difference in glucose yield from the samples was not in accordance with that of glucose concentration. The glucose yield of the CCR without pretreatment was 70.6% after 96 h of enzymatic hydrolysis, which just a littler lower than that of the sample treated with neutral sulfite (74.5%). The ethanol sulfite sample had the highest glucose yield (81.2%) in the saccharification samples, which was over 10% higher than that of the raw material. The acidic sulfite pretreated sample shared a similar glucose yield (78.2%) with the CCR treated with alkaline sulfite (79.4%).

It is well known that the physical barrier and non-productive binding to enzyme of lignin is the main problem in CCR enzymatic hydrolysis [22,23]. After the sulfite pretreatment, part of the lignin had been removed and reduced the barrier of lignin to cellulose enzymatic hydrolysis [24,25]. More importantly, the sulfonation and degradation of the lignin increased its hydrophilism, which may be favorable to reduce the non-productive binding between lignin and cellulase [7]. Among the four kinds of sulfite pretreatment, the ethanol and alkaline sulfite pretreatment had similar amounts of lignin removal, but the glucose yield of the ethanol sulfite sample was higher than that of the alkaline sulfite treated sample. It may be attributed to the fact that more hydrophilic groups had been introduced in the CCR after ethanol sulfite pretreatment [26]. Moreover, the glucose yield from the acidic sulfite sample was higher than that from neutral when they shared similar lignin proportion. This phenomenon was closely related to the enhanced hydrophily of lignin during the sulfite pretreatment.

The glucose yield of the pretreated samples based on the cellulose in raw material (GPR) can be obtained from the saccharification efficiency together with solid yield in pretreated progresses. All pretreated samples had higher GPR than the raw material expect the alkaline sulfite sample. It should be attributed to the serve degradation of cellulose during the alkaline sulfite pretreatment. The sample treated with ethanol sulfite had the highest GPR (79.34% after 96 h of enzymatic hydrolysis). However, if the soluble lignin had been rationally used, it would be a good choice to adopt these pretreatment, especially the ethanol sulfite pretreatment, which not only can increase the glucose yield of CCR, but also results in a certain amount of lignin solution. Furthermore, the residual of cellulosic hydrolysis may have a good application prospect, as the lignin had been sulfonated during the sulfite pretreatments.

The sulfites pretreatments had enhanced the lignin sulfonation which can not only be benefited to the cellulosic hydrolysis but also bring a good application prospect for lignin (both degraded and residual). So from the whole processes of the biorefineries, the sulfite pretreatment may be a good choice. Moreover, the ethanol sulfite pretreatment presented an appealing effect.

### Conductometric titrations

**Figure 3:**
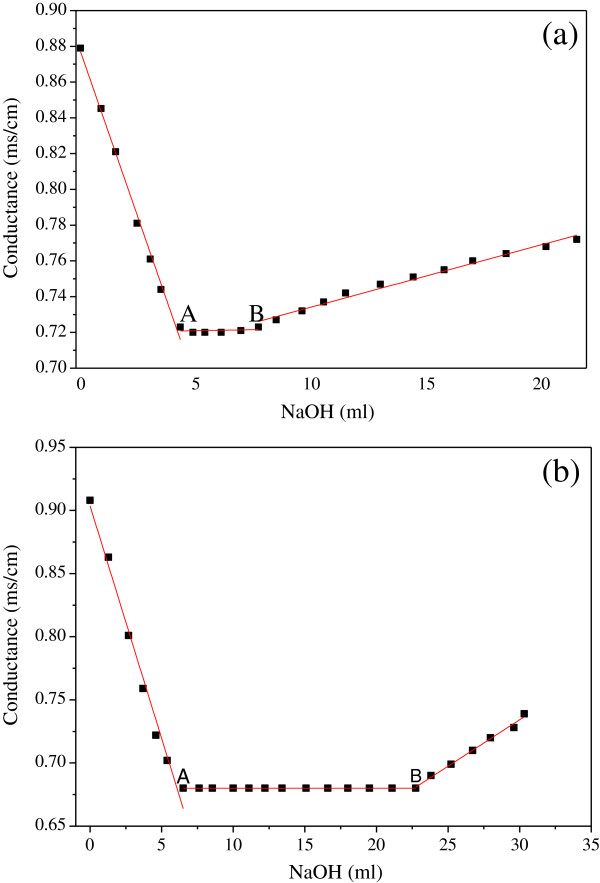
**Conductivity titration curve of raw material (a) and the ethanol sulfite pretreated sample (b).** “A” represents the equivalent point of strong acid, while “B” represents the equivalent point of weak acid groups.

The hydrophility of lignin is intimate connection with its hydrophilic groups. So, in this study, the sulfonic group and weak acid groups of the samples had been measured by conductometric titration. The conductivity titration curves of the raw material and the ethanol sulfite pretreated sample were shown in Figure 3a and Figure 3b, respectively.

The conductometric titration is based on changes in conductance of the suspension. The resultant conductivity of the suspension was plotted against the volume of alkali added. So, before the equivalent point “A,” the alkali was used to neutralize the hydrogen from HCl added before the titration as well as the sulfonic group in the sample. The content of sulfonic group was the difference between the alkali required to reach the inflection point “A” and the HCl added before the titration. The weak acid groups were calculated using the volume of alkali required to reach the second inflection point “B” from the first inflection point “A” of the plot [27,28]. And, the total amount of hydrophilic groups was the sum of the sulfonic group and the weak acid groups.

**Figure 4:**
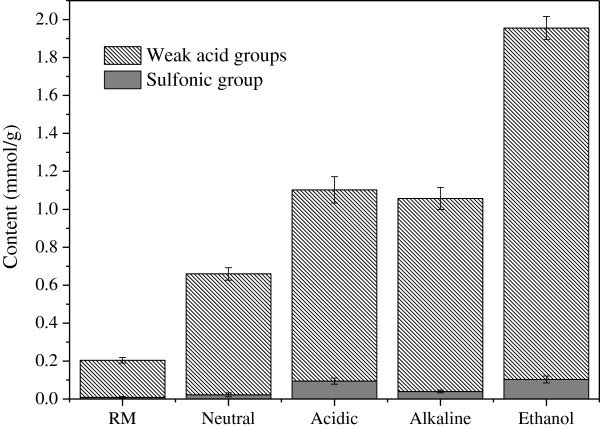
**Variation of sulfonic group and weak acid groups after sulfite pretreatments.** RM represents the raw material of corn cob residue, while Neutral, Acidic, Alkaline and Ethanol represent corn cob residue treated with sulfite in neutral, acidic, alkaline and ethanol environment, respectively.

The conductivity titration curves of other samples were similar with that of the ethanol sulfite pretreated sample, but the volumes of alkali required to reach the two inflection points “A” and “B” were different, which implied that the amount of sulfonic group and weak acid groups in these samples was different (Figure 4). In the raw material, there was little sulfonic group that may have been introduced during the furfural production with dilute sulphuric acid hydrolysis. And, the amount of weak acid groups was 0.196 mmol/g. The sulfite pretreatments increased the amount of both the sulfonic group and the weak acid groups. The nucleophilic performance of the active agents determines the degree of lignin sulfonation and degradation. During the neutral sulfite pretreatment, the nucleophilic reagents were SO_3_^ 2-^ and HSO_3_^ -^, which attracted the ether linkage in lignin resulting in its break and the introduction of the sulfonic acid group. So, the sulfonic group as well as the weak acid groups increased to 0.022 mmol/g and 0.637 mmol/g, repectively, after neutral sulfite treated. Compared with the neutral sulfite pretreatment, the nucleophilic reaction during sulfite treatment in the acidic and alkaline environments was catalyzed by the hydrogen or hydroxyl ions, which was beneficial to the lignin sulfonation and degradation, and their weak acid groups were similar (1.01 mmol/g, acidic; 1.02 mmol/g, alkaline). However, the dissolubility of lignin in the alkaline environment was higher than that in the acidic environment, so the lignin after sulfonate was much easier to dissolve in aqueous alkali, causing lower sulfonic acid group in the alkaline sulfite treated sample (0.039 mmol/g) than in the acidic sample (0.094 mmol/g). The ethanol sulfite treated sample showed the highest sulfonic group (0.103 mmol/g) and weak acid groups (1.85 mmol/g) in all sulfite treated samples. As explained above, the ethanol sulfite pretreatment was beneficial to solution penetration and its reaction with lignin. And, the ethanol may not only provide an alcohol solution environment but also take part in the reaction with lignin. The hydroxyl may have grafted to lignin and increased its hydrophilicity. The increased hydroxyl provided convenience for sulfonation reaction [29], which improved the solubility and amount of removed lignin. The increase in these hydrophilic groups reduced the non-productive binding between lignin and cellulase and enhanced the glucose yield of the ethanol sulfite pretreated sample.

### FT-IR spectra

**Figure 5:**
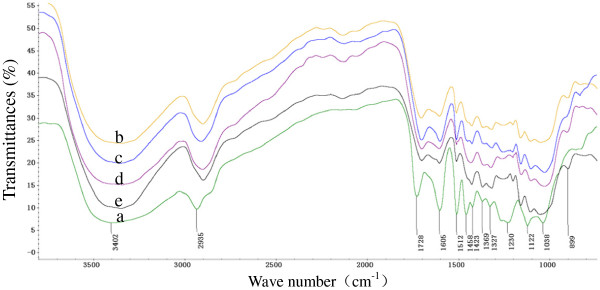
**FT-IR spectra of samples.** ‘a’ represents the raw material of the corn cob residue, while ‘b’, ‘c’, ‘d’, and ‘e’ represent the corn cob residue treated with sulfite in neutral, acidic, alkaline, and ethanol environments, respectively.

FT-IR spectroscopy provides information about chemical composition, molecular conformation, and hydrogen bonding patterns of cellulose allomorphs [30]. The FT-IR spectra of the CCR samples are shown in Figure 5.

The analyses of the recorded spectra of the lignin samples used in this study were all based upon the assignments given by previous investigations [31,32]. As can be seen from the spectra, all the samples showed broad bands at 3410–3460 and 2897–2905 cm^-1^, attributed to the stretching of –OH groups and to C–H stretching, respectively, corresponding to the aliphatic moieties in cellulose, and survived lignin. The band around 1715 cm^-1^ in the spectra, assigned to carbonyl/carboxyl stretching, indicates the existence of the hydrophilic groups in the samples. The bands at 1605 and 1512 cm^-1^, which are attributed to the skeletal and stretching vibration of benzene rings, became weak in spectra of the sulfite treated samples because of lignin removal during the sulfite pretreatments. The disappearance of the band at 1458 cm^-1^ (bending vibration of the methoxyl on benzene rings) in the spectra of the samples after pretreatments implied the methoxyl in the lignin had been removed during the sulfite pretreatments. Moreover, this conclusion was confirmed by the variation of the bands at 1270 and 1230 cm^-1^, which are attributed to the aromatic core of guaiacyl and syringyl, respectively. C–H bending occurs at 1370 (1368) cm ^-1^, and the C–C (C–O) vibration absorption appears at 1328 cm^-1^. The main differences in the spectra after sulfite pretreatment were the bands at 1168 and 1190 cm^-1^ (attributed to the absorption of sulfonic group), which were not obvious in the spectrum of the raw material. Furthermore, the fact that the bands were stronger in the spectrum of the ethanol sulfite pretreated sample than the other treated samples indicates that the sulfonation degree of the ethanol sulfite treated sample was the highest, which was according with the result of conductivity titration.

Two absorption bands around 1119 and 899 cm^-1^ arise from C–O–C stretching at the β-(1–4)-glycosidic linkages [33]. Strong peaks at 1056 (1058) and 1038 cm^-1^ are indicative of C–O stretching at C-3 and C–C stretching and C–O stretching at C-6 [34]. The most important bands that helped to identify the cellulose component are at 1042 cm^-1^, attributed to amorphous cellulose and crystallized cellulose II, and at 1430 cm^-1^, attributed to crystallized cellulose I. The band around 1427 cm^-1^ in the spectra of sulfite treated samples indicated a mixed structure of crystallized cellulose I and amorphous cellulose in all the samples [35]. However, the band of the untreated sample appearing at 1423 cm^-1^ may imply that the crystallized areas of cellulose in the CCR increased after the sulfite pretreatments. The FT-IR spectra analysis further confirmed that the lignin of the CCR had been removed partly and the lignin had been sulfonated after the sulfite pretreatments. These variations were beneficial to its enzymatic hydrolysis.

## Conclusion

The lignin in the CCR (43.2%) reduced to 37.8%, 38.0%, 35.9%, and 35.5% after the sulfite pretreatment in neutral, acidic, alkaline, and ethanol environments, respectively. Meanwhile, the glucan of the CCR increased from 48.1% to 51.6%, 51.8%, 49.3%, and 55.5%, respectively after the corresponding sulfite pretreatment. These results indicate that the ethanol sulfite pretreatment is the best method for lignin removal and has the least cellulose degradation among the selected sulfite methods. The glucose yield (based on the cellulose in the treated sample) of the ethanol sulfite sample was the highest (81.2%) among the saccharification samples, which was over 10% higher than that of the raw material (70.6%). In the raw material, there was a little sulfonic group and weak acid groups, which increased after sulfite pretreatments. Meanwhile, the ethanol sulfite treated sample had the highest sulfonic group (0.103 mmol/g) and weak acid groups (1.85 mmol/g) in all the sulfite treated samples. These variations enhance the hydrophilicity of the samples, which may improve the glucose yield of the samples. In the FT-IR spectra, the variation of bands at 1168 and 1190 cm^-1^ (attributed to the absorption of the sulfonic group) confirmed lignin sulfonation during sulfite pretreatment. The disappearance of the band 1458 cm^-1^ in the spectra of the samples after pretreatments implied that the methoxyl in the lignin had been removed during the sulfite pretreatments. So, during the sulfite pretreatments, the lignin in the CCR can be degraded and sulfonated, and the pretreatments improve the hydrophility and enhance the glucose yield of the material. Moreover, the ethanol sulfite pretreatment with higher lignin removal and glucose yield is slightly better than the other sulfite pretreatments.

## Methods

### Corn cob residue

The corn cob residue (CCR) produced from corn cob was kindly supplied by the Chunlei Furfural Corporation (Hebei, China). The residues, which had a pH of 2 to 3 initially, were immersed in the fresh water for 24 h and then washed with distilled water until neutral to remove acid, furfural and other toxic products to enzyme and yeast. Before milled to a size under 40 meshes, CCR should be dry at 50°C for 12 h. And then the dried materials were stored in sealed bags at room temperature until further processing.

### Sulfite pretreatments

The pretreatments were performed in the pressure bottles with screw cap (Synthware Co., Ltd). During neutral sulfite pretreatment, the sodium sulfite (1%, w/v) were added in the bottles with CCR slurry in water (10%, w/v); additionally no other more chemicals was used, and the final pH was 7.5 at normal temperature. When the CCR (10%, w/v) was dispersible in dilute acidic solution (0.5% H_2_SO_4_, w/v) before the sodium sulfite (1%, w/v) added, this process was defined as acidic sulfite pretreatment (pH 2.3). The alkaline sulfite pretreatment (pH 12.4) was operated similarly as the acidic sulfite pretreatment, but the dilute acidic solution had been replaced by dilute alkali solution (0.5% NaOH, w/v) to provide alkaline environment. The ethanol sulfite pretreatment was slightly different, and 1% sodium sulfite (w/v) was added in the bottle with CCR (10%, w/v) dispersed in alcohol solution (80%, v/v), with pH 7.9.

The screw caps of the bottles were tightened after the sodium sulfite had been added. Then the bottles were placed in the water bath shaker with 100 rpm at 80°C for 3 h. The mixture in the bottles after pretreatment was filtered to separate the solid residues and the filtrate fraction. The solid residues were thoroughly washed with tap water to neutral pH, then vacuum dried at 50°C, and finally weighed. The pretreated samples after vacuum dry were ground into powder with mortar before analysis. The lignin and glucan contents of CCR as well as pretreated samples were analyzed according to the National Renewable Energy Laboratory (NREL) methods [36].

### Enzymatic saccharification

The solid residues obtained from the pretreatment were further submitted to enzymatic hydrolysis. Each enzymatic hydrolysis treatment was preformed at 47°C, pH 4.8 with a substrate concentration of 2.5% (w/v). Cellulase (Celluclast 1.5L, 74FPU/ml, Sigma Co., St. Louis, MO) loading for the CCR was 12 FPU/g-cellulose and the β-glucosidase (Novozyme 188, 175 CBU/ml, Sigma Co., St. Louis, MO) loading was 15 CBU/g-cellulose. The hydrolysis of CCR without pretreatment was performed as control. The saccharification was cultivated on a rotary shaker at 180 rpm for 96 h. Samples were withdrawn and centrifuged at 10000×g for 5 min. The hydrolysates were filtered through 0.2 um filters and diluted properly for further neutral sugar analysis.

### Analysis

The neutral sugars during saccharification process were analyzed by HPLC (Waters 2695e, USA) with Aminex HPX-87P (300×7.8 mm, Bio-Rad, USA) at 85°C and refractive index detection detector at 35°C. The injection volume of the sample was 10 μL, and distilled water was used as the eluent, at a flow rate of 0.6 ml/min. The glucose yield was calculated assuming that 1 g cellulose present in the liquid theoretically gave 1.11 g of glucose. Assays were performed in 3 repeated experiments, and the mean values are calculated.

The conductometric titrations were used to detect the content of weak acid groups and sulfonic group in the samples. Before the conductometric titrations, the ground CCR samples were converted to their fully protonated form by soaking the samples at 1% consistency in 0.01 M hydrochloric acid for 16 h. The samples with pH close to 2.2 after 16 h of soaking were then vacuum-filtered using a Buchner funnel and washed several times with deionized water until the pH of the water filtrate was close to 6.0. The vacuum was maintained until no more water could be extracted from the CCR samples. Approximately 0.5 g of the protonated CCR sample was dispersed in 1 mM sodium chloride (100 ml) and addition of 0.5 ml of 0.05M HCl was made before the start of titration. The titration was performed with 5 mM NaOH in a constant temperature water bath set at 25°C. The conductivity meter (DDSJ-308A, Shanghai Precision & Scientific Instrument Co. Ltd.) was exploited to detect the variation of the conductance during the titration [27]. The content of sulfonic groups (SG) and weak acid groups (WAG) were calculated according to the following formulas:

$$\begin{array}{l} SG=\left(c_{2}*V_{2}-c_{1}*V_{1}\right)/m\left(\text{m mol}/g\right);WAG\\ \;=\left(c_{2}*V_{3}-c_{2}*V_{2}\right)/m\left(\text{m mol}/g\right)\text{.} \end{array}$$

In which, *c*_*1*_ is the concentration of HCl solution (mol/L); *V*_*1*_ is the volume of HCl solution addition (ml); *c*_*2*_ is the concentration of NaOH solution (mol/L); *V*_*2*_ is the consumed volume of NaOH solution before first equivalent point (ml); *V*_*3*_ is the consumed volume of NaOH solution before second equivalent point (ml); *m* is quality of the tested sample.

The FT-IR spectra of ground samples the lignin fractions were obtained on a Nicolet-750 FT-IR spectrophotometer using KBr discs containing 1% finely ground samples in the range of 4000–400 cm^-1^.

## Abbreviations

CCR: Corn cob residue; GPR: Glucose yield of the pretreated samples based on the cellulose in raw material; NREL: National Renewable Energy Laboratory; SG: Sulfonic groups; WAG: Weak acid groups.

## Competing interests

The authors declare that they have no competing interests.

## Authors’ contributions

BL and JJ designed and coordinated the study; BL, XY and GY carried out the experiments; BL and YH analyzed the results; BL and XY wrote the paper; JJ and YH reviewed the paper. All authors read and approved the final manuscript.
